# Usp9X Regulates Cell Death in Malignant Peripheral Nerve Sheath Tumors

**DOI:** 10.1038/s41598-018-35806-5

**Published:** 2018-11-26

**Authors:** E. Bianchetti, S. J. Bates, S. L. Carroll, M. D. Siegelin, K. A. Roth

**Affiliations:** 1 Department of Pathology & Cell Biology, Columbia University Vagelos College of Physicians and Surgeons, New York, USA; 2 Medical University of South Carolina, Department of Pathology and Laboratory Medicine, Charleston, South Carolina USA

## Abstract

Malignant peripheral nerve sheath tumors (MPNSTs) are the leading cause of death in neurofibromatosis type 1 (NF1) patients. Current treatment modalities have been largely unsuccessful in improving MPNST patient survival, making the identification of new therapeutic targets urgent. In this study, we found that interference with Usp9X, a deubiquitinating enzyme which is overexpressed in nervous system tumors, or Mcl-1, an anti-apoptotic member of the Bcl-2 family whose degradation is regulated by Usp9X, causes rapid death in human MPNST cell lines. Although both Usp9X and Mcl-1 knockdown elicited some features of apoptosis, broad spectrum caspase inhibition was ineffective in preventing knockdown-induced MPNST cell death suggesting that caspase-independent death pathways were also activated. Ultrastructural examination of MPNST cells following either Usp9X interference or pharmacological inhibition showed extensive cytoplasmic vacuolization and swelling of endoplasmic reticulum (ER) and mitochondria most consistent with paraptotic cell death. Finally, the Usp9X pharmacological inhibitor WP1130 significantly reduced human MPNST growth and induced tumor cell death in an *in vivo* xenograft model. In total, these findings indicate that Usp9X and Mcl-1 play significant roles in maintaining human MPNST cell viability and that pharmacological inhibition of Usp9X deubiquitinase activity could be a therapeutic target for MPNST treatment.

## Introduction

Neurofibromatosis type 1 (NF1) is a genetic neurocutaneous disease with an incidence of 1:3000^1,2^ characterized by a predisposition to multiple peripheral nerve sheath tumors^3^. The vast majority of NF1-associated nerve sheath tumors are benign, but malignant peripheral nerve sheath tumors (MPNSTs) are the leading cause of death in NF1 patients. MPNSTs are aggressive Schwann cell-derived soft tissue sarcomas and occur in 5 to 10% of patients with NF1^4^. Approximately half of MPNSTs are associated with NF1 and often arise from benign plexiform neurofibromas^5^. Currently, standard MPNST therapy is tumor resection with wide surgical margins, but patient prognosis is poor due to variables such as tumor size, anatomic location, propensity to metastasis and limited tumor cell sensitivity to chemotherapy and radiation^1^. Therefore, identification of new therapeutic targets to treat this aggressive neoplasm is a high clinical priority.

Usp9X is a deubiquitinating enzyme which is overexpressed in various human cancers, including nervous system tumors, such as glioblastoma (GBM)^6^. Genetic and/or pharmacological inhibition of Usp9X activity has been shown to induce tumor cell death in both *in vitro* and *in vivo* models of GBM^6–8^. Previous studies have demonstrated that down-regulation of Usp9X is followed by enhanced degradation of the anti-apoptotic Bcl-2 family member, myeloid cell leukemia 1 (Mcl-1)^7,9^. Furthermore, Mcl-1 down-regulation is known to be an important determinant of apoptosis in sarcomas^10^. Our findings suggest that Usp9X and Mcl-1 are novel targets for the treatment of MPNSTs and that paraptosis, a caspase-independent type of regulated cell death, may play a role in MPNST cell death induced by Usp9X inhibition.

## Results

### Usp9x is expressed in human MPNST cell lines

Usp9X expression in MPNSTs has not previously been reported. Thus to ensure potential human clinical relevance, we first examined Usp9X expression levels in a panel of human MPNST cell lines (Suppl. Figure 1a). All MPNST cells showed Usp9X protein expression, albeit at different levels. The results confirm that the Usp9X protein is expressed in MPNST cells, reinforcing the notion that Usp9X is a viable, potential therapeutic target for MPNST.

### Usp9X inhibition causes massive reduction in MPNST cell viability

**Figure 1:**
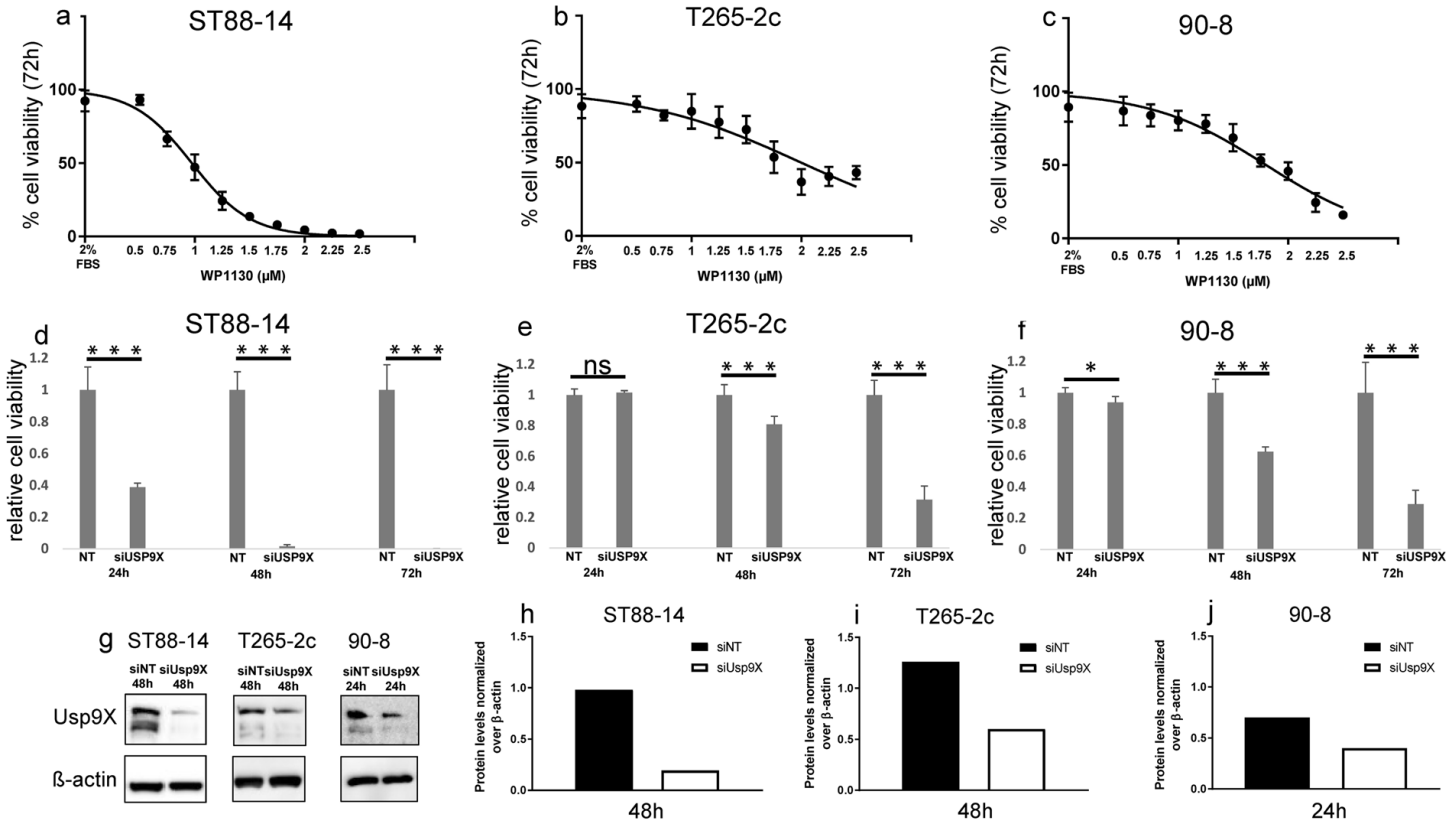
Usp9X inhibition causes massive reduction in cellular viability in MPNST cell lines. (**a–c**) ST88-14 (**a**), T265-2c (**b**), 90-8 cells (**c**) were treated with increasing concentrations of WP1130 for 72 h. Cellular viability was determined by CellTiter-Glo assay and the IC_50_-values were calculated based on a non-linear regression analysis. Data are presented as mean and SD, n = 3. (**d**–**f**) Usp9X was silenced in ST88-14 (**d**), T265-2c (**e**), 90-8 cells (**f**) with non-targeting (NT) RNA or siUsp9X for 24, 48 and 72 h. Cellular viability was determined by CellTiter-Glo assay and relative cell viability was calculated. Data are presented as mean and SD, n = 3. * = 0.0129 *** ≤ 0.001. (**g**–**j**) Usp9X knock down was performed in ST88-14, T265-2c and 90-8 cells. Whole-cell extracts were examined by Western blot for Usp9X. ß-actin Western blot analysis was performed to confirm equal protein loading. Bar graphs show protein quantification analyzed through ImageJ. Data are presented as mean and SD, n = 3.

To investigate the potential role of Usp9X in regulating MPNST cell survival, we first examined the effects of inhibiting Usp9X enzymatic activity with the deubiquitinase inhibitor, WP1130, a pharmacological inhibitor of Usp9X known also as Degrasyn^6^, on three NF1 patient-derived MPNST cell lines (ST88-14, T265-2c and 90-8). WP1130 caused a concentration-dependent decrease in cell viability after 72 h in all three cell lines, with ST88-14 cells being particularly sensitive (Fig. 1a,b,c). In these experiments, we used a concentration range between 0.5 and 2.5 µM, established from preliminary results (Suppl. Figure 1b,c). In addition to Usp9X, WP1130 inhibits the enzymatic activity of multiple deubiquitinases; thus, to more selectively determine the effects of Usp9X inhibition on MPNST cell survival *in vitro*, we silenced Usp9X gene using siRNA. Suppression of Usp9X was confirmed by immunoblotting (Fig. 1g–j). In all three cell lines, Usp9X gene silencing resulted in a significant reduction in viable cell numbers after 72 h (Fig. 1d–f). Consistent with the results obtained with pharmacological inhibition of Usp9X activity, ST88-14 cells were most strongly affected by Usp9X silencing, with a significant reduction in cell viability observed after just 24 h treatment (Fig. 1d). These data support the conclusion that Usp9X is critical for the survival of MPNST cells.

### Usp9X inhibition depletes Mcl-1

Based on our previously published observation that Usp9X induces a decline of Mcl-1 protein levels in GBM cells^9,11^, we hypothesized that similar effects would be observed in MPNST cells. To test this hypothesis, ST88-14 cells were treated with WP1130 at two different concentrations (Suppl. Figure 1e) and Mcl-1 levels were assessed by Western blot after 24 and 48 h. Usp9X interference decreased endogenous Mcl-1 protein levels at both concentrations after treatment for 48 h (Suppl. Figure 1e). Consistent with these results, Usp9X knock-down in ST88-14 cells resulted in decreased Mcl-1 levels at 48 h as well (Suppl. Figure 1d).

### Usp9X knock-down causes MPNST cell death with variable caspase activation and features of apoptosis

**Figure 2:**
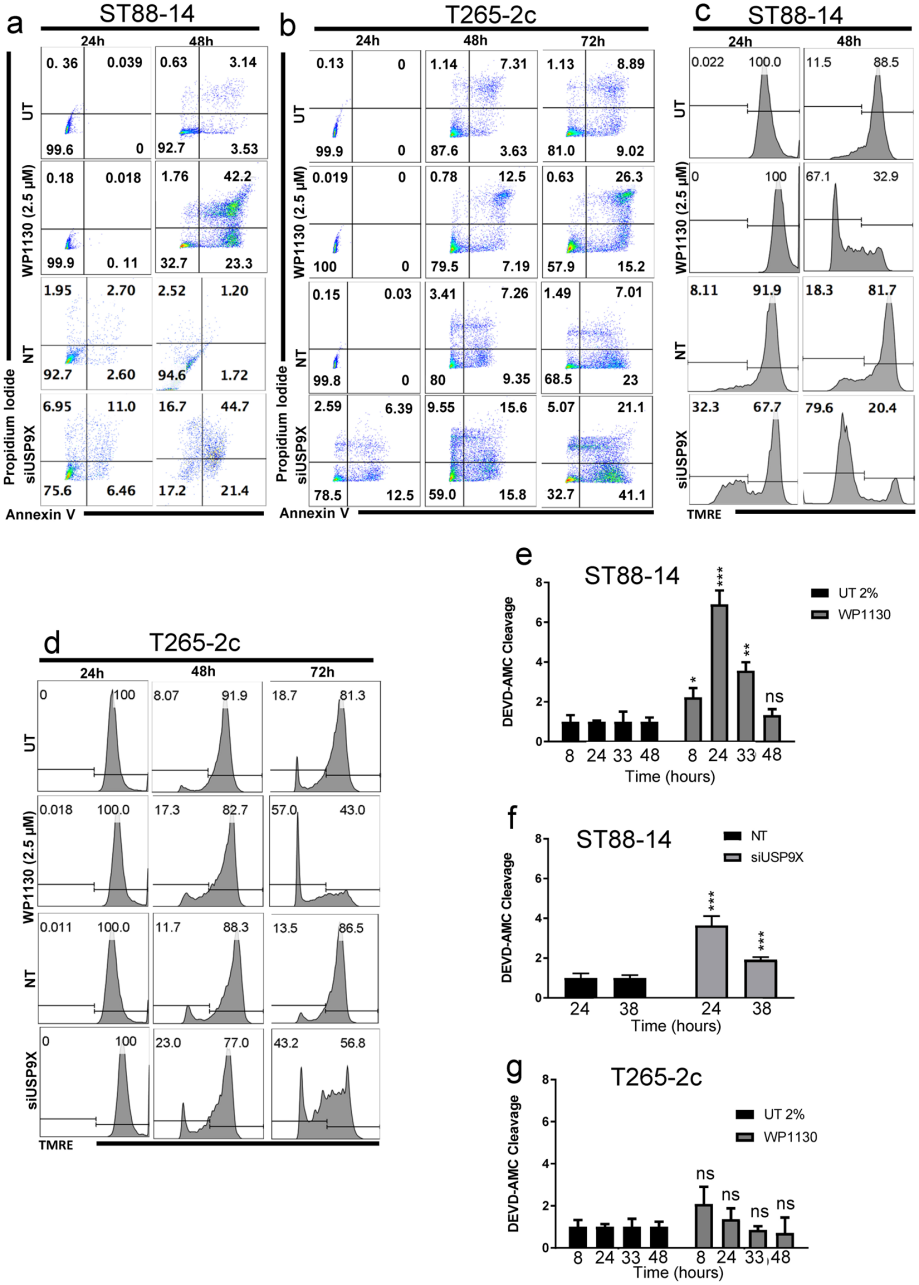
Usp9X inhibition induces cell death with features of apoptosis and loss of membrane potential in MPNST cell lines. (**a**–**d**) ST88-14 (**a**,**c**) and T265-2c cells (**b**,**d**) were treated with WP1130 at the concentration of 2.5 µM, or transfected either with non-targeting (NT)-siRNA or Usp9X-siRNA. (**a**,**b**) Staining for annexin V/Propidium Iodide was performed prior to flow cytometric analysis. Representative flow plots are shown. N = 3. (**c**,**d**) Cells were stained with Mitochondrial Membrane Potential Assay Kit (TMRE), then flow cytometric analysis was performed. N = 3. (**e**,**f**) ST88-14 cells were treated with WP1130 at the concentration of 2.5 µM (**e**) or transfected either with non-targeting (n.t.)-siRNA or Usp9X-siRNA (**f**), then caspase 3-like enzymatic activity was measured at different time points. Column: mean. Error bar: SD, n = 3. * = 0.0209 ** = 0.0026 *** ≤ 0.001. (**g**) T265-2c cells were treated with WP1130 at the concentration of 2.5 µM prior to caspase 3-like activity measurement at different time points.

To determine if Usp9X inhibition induced apoptosis of MPNST cells, we inhibited Usp9X pharmacologically with WP1130 and silenced it by siRNA, as well, in ST88-14 and T265-2c cell lines and examined various biochemical indicators of apoptosis. Usp9X pharmacological inhibition resulted in several features of apoptosis (Fig. 2a,b), including a time-dependent decrease in mitochondrial membrane potential (Fig. 2c,d), in both cell lines. Consistent with the viability data, ST88-14 cells were more rapidly and strongly affected by Usp9X inhibition, than T265-2c cells. As with pharmacological inhibition of Usp9X activity, Usp9X knock-down produced biochemical features of apoptotic cell death including a significant loss in mitochondrial membrane potential (Fig. 2a–d). ST88-14 cells consistently showed more dramatic effects after Usp9X interference, compared to T265-2c cells (Fig. 2a–d) and caspase 3-like enzymatic activity was more pronounced after Usp9X knock-down or WP1130 treatment in ST88-14 cells compared to T265-2c cells (Fig. 2e–g).

### Interference with Mcl-1 produces cell death with apoptotic features in MPNST cell lines

**Figure 3:**
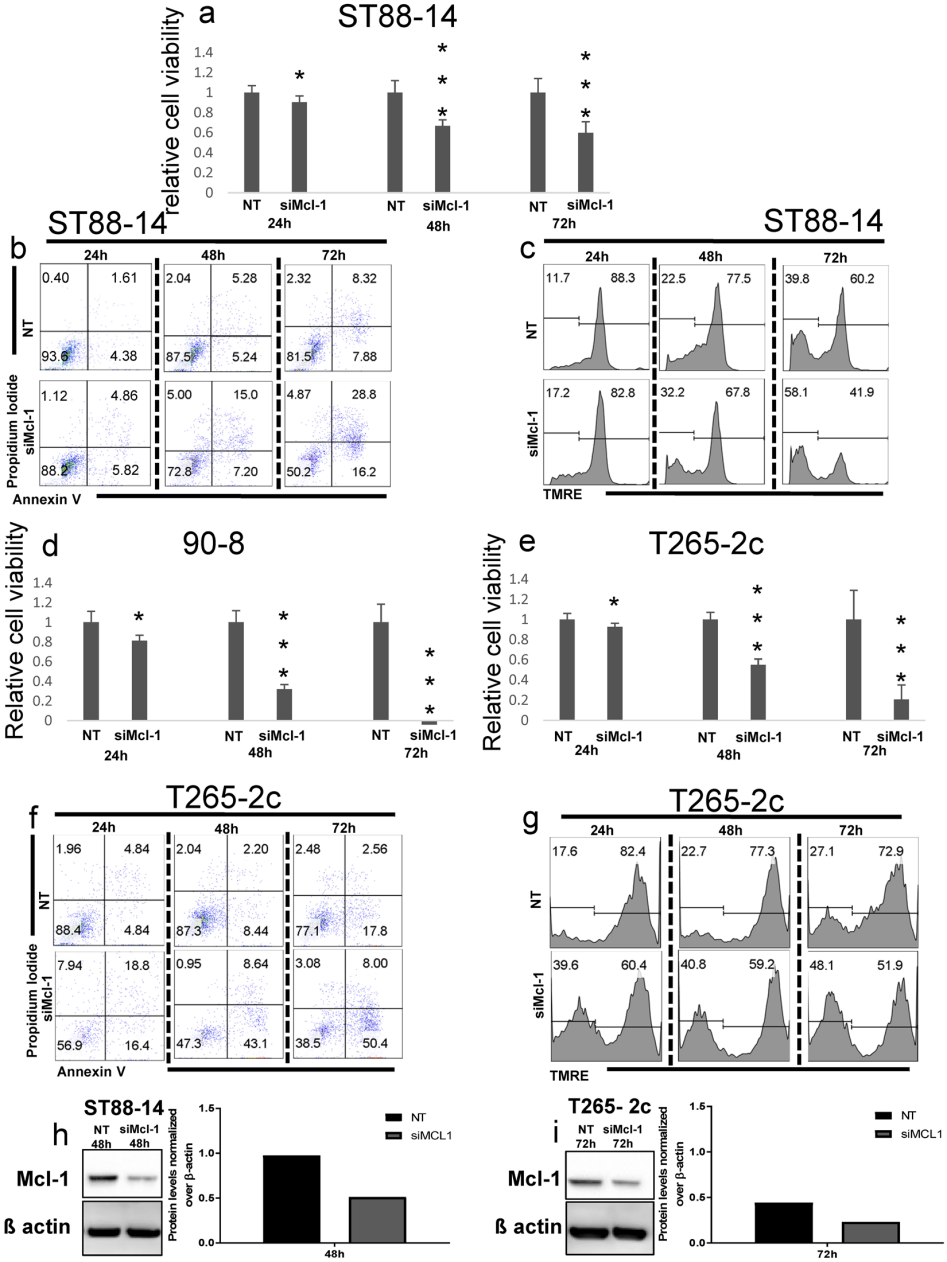
Mcl-1 knock-down causes massive reduction in cellular viability, cell death with features of apoptosis and loss of membrane potential in MPNST cell lines. (**a**,**d**,**e**) ST88-14 (**a**) 90-8 (d), T265-2c (**e**) cells were transfected either with non-targeting (NT)-siRNA or Mcl-1-siRNA for 24, 48 and 72 h. Cellular viability was determined by CellTiter-Glo assay and relative cell viability was calculated. Data are presented as mean and SD, n = 3. *(**a**) = 0.0161, *(**d**) = 0.0015, *(**e**) = 0.0168, *** ≤ 0.001 (**b**,**f**) Staining for annexin V/Propidium Iodide was performed prior to flow cytometric analysis. Representative flow plots are shown. N = 3. (**c**,**g**) Staining for Mitochondrial Membrane Potential Assay Kit (TMRE) was performed prior to flow cytometric analysis. Representative flow plots are shown. N = 3. (**h**,**i**) Knock down of Mcl-1 was confirmed in ST88-14 cells (**h**) and T265-2c cells (**i**) by Western blot analysis. ß-actin was used to ensure equal loading.

Usp9X regulates cell survival through both Mcl-1-dependent and –independent processes. To determine if Mcl-1 is a regulator of MPNST survival, we examined the effects of Mcl-1 knockdown on the three MPNST cell lines. As with Usp9X pharmacological inhibition or knock-down, Mcl-1 knock-down resulted in significant cell death in all cell lines tested (Fig. 3a–g), most prominently in T265-2c and 90-8 cell lines (Fig. 3d–g). All three cell lines showed time-dependent apoptotic features with enhanced DNA fragmentation, annexin V staining (Fig. 3b,f), and loss in mitochondrial membrane potential (Fig. 3c,g) with maximal response at 72 h after transfection. T265-2c cells were more sensitive to Mcl-1 depletion (Fig. 3d,e), compared to ST88-14 cells (Fig. 3a), with strong features of apoptosis (Fig. 3f,g). Suppression of Mcl-1 was confirmed by immunoblotting (Fig. 3h,i). Consistent with these results, pharmacological inhibition of Mcl-1 with A-1210477^12^, caused a concentration-dependent decrease in cellular viability after 72 h in all tested cell lines (Suppl. Figure 2). These data indicate that in addition to being sensitive to Usp9X inhibition, MPNST cell lines are also susceptible to Mcl-1 loss, which appears to activate apoptosis, most prominently in the T265-2c cell line.

### Caspase-inhibition provides minimal protection from cell death after Usp9X or Mcl-1 depletion

**Figure 4:**
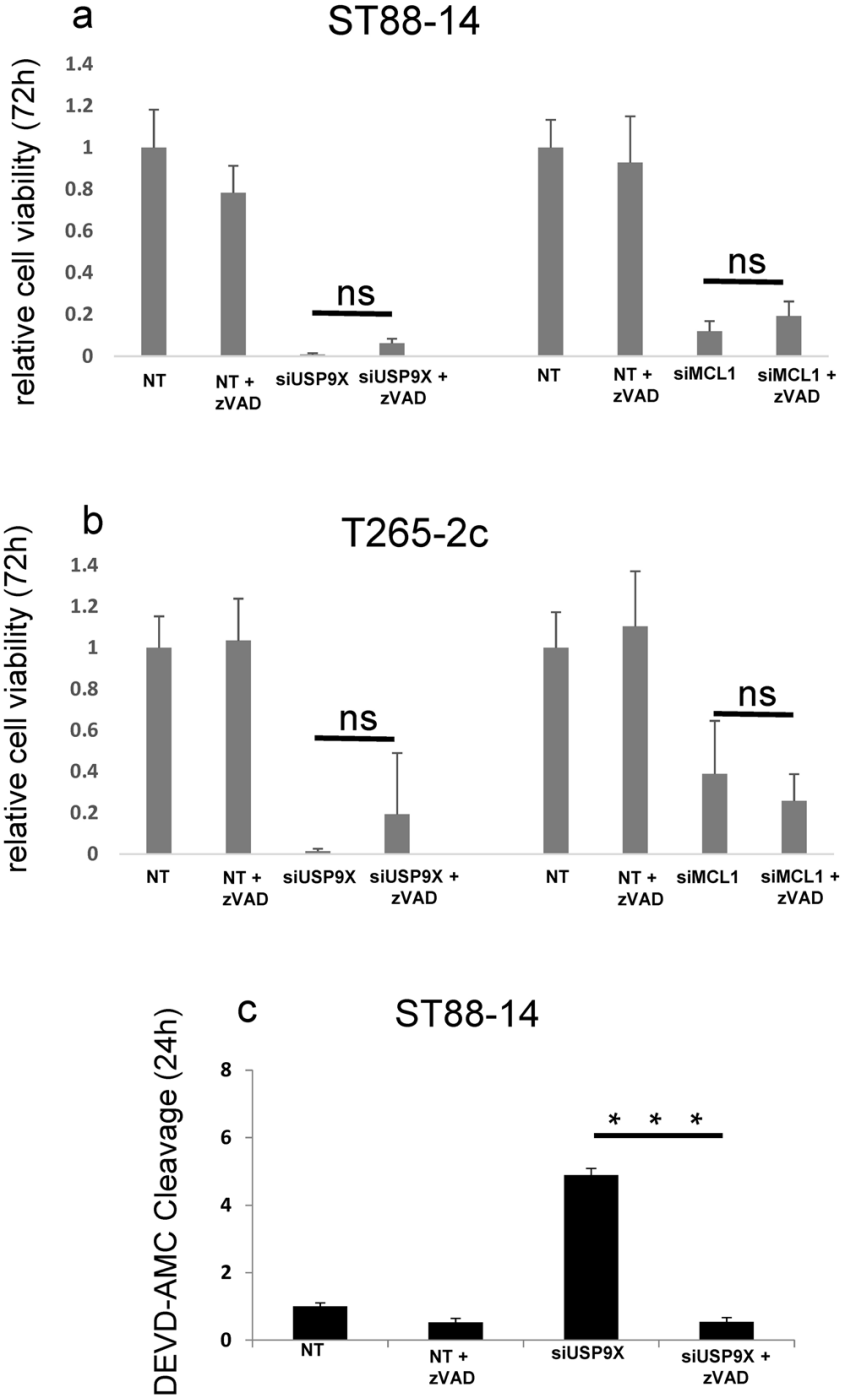
Caspase-dependent apoptosis is not the exclusive MPNST cell death pathway activated after Usp9X and Mcl-1 knock down *in vitro*. (**a**,**b**) ST88-14 (**a**) and T265-2c (**b**) cells were transfected with non-targeting (NT)-siRNA with or without z-VAD as controls, or Usp9X-siRNA, or Mcl-1-siRNA for 72 h. Cellular viability was determined by CellTiter-Glo assay and relative cell viability was calculated. Data are presented as mean and SD, n = 3. (**c**) Caspase-3 like activity was measured at 24 h after treatments in ST88-14 cell line. Data are presented as mean and SD, n = 3.

To determine if caspase-dependent apoptosis is the main cell death pathway activated by Usp9X or Mcl-1 depletion, we tested the ability of ZVAD, a broad-spectrum caspase inhibitor, to inhibit Usp9X and Mcl-1 knock-down induced MPNST cell death. Despite producing a complete inhibition of caspase 3-like enzymatic activity (Fig. 4c), ZVAD failed to significantly attenuate cell death after Usp9X or Mcl-1knock-down at 72 h in ST88-14 (Fig. 4a) or T265-2c cells (Fig. 4b), indicating that other caspase-independent death pathways are likely activated in parallel with apoptosis following Usp9X or Mcl-1 inhibition.

### Usp9X inhibition causes ER stress in MPNST cell lines

**Figure 5:**
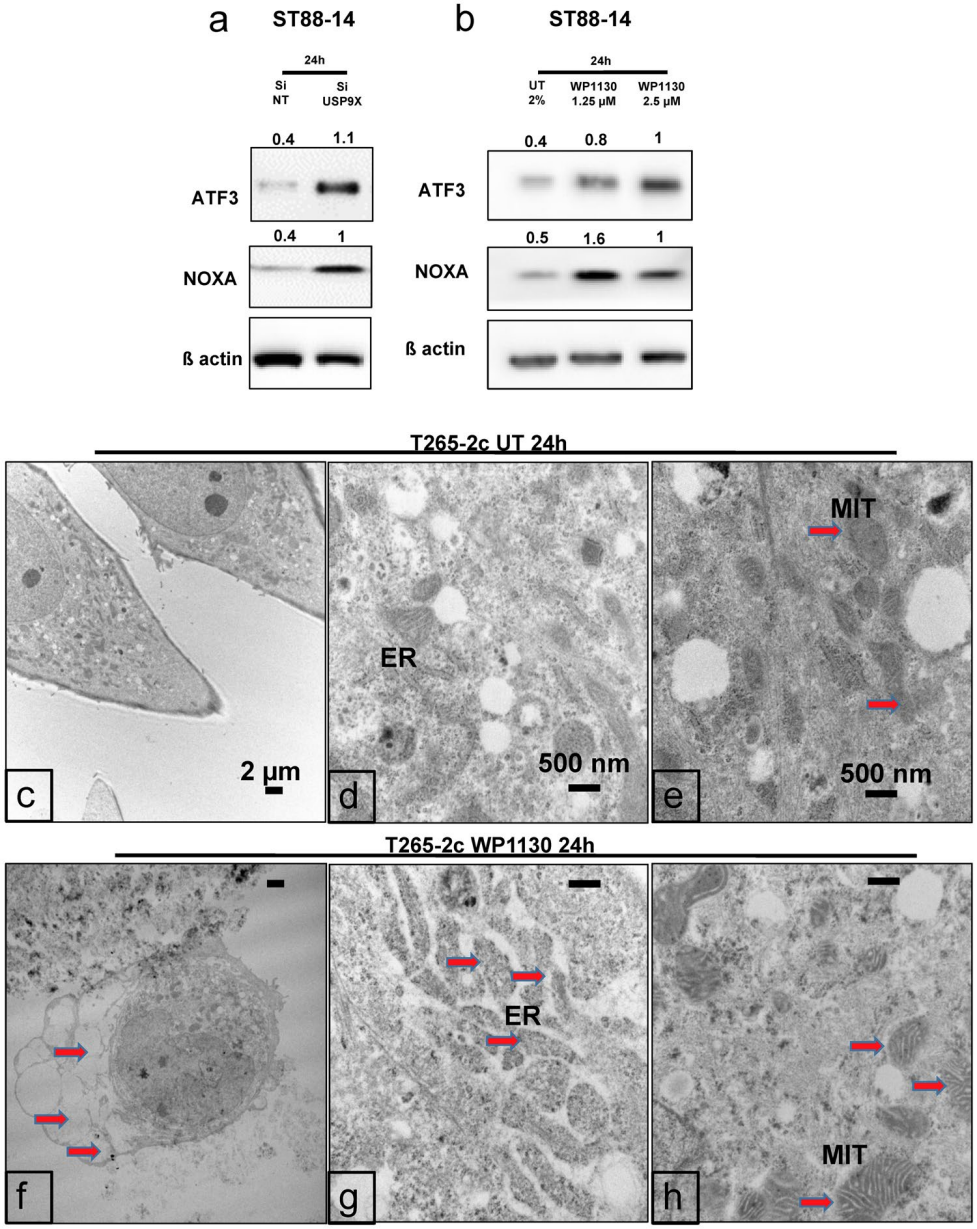
Usp9X inhibition causes Noxa increase and ER stress in MPNST cell lines. Ultrastructural analysis shows features of paraptosis. (**a**,**b**) ST88-14 cells were transfected for 24 h with either non-targeting (NT)-siRNA or Usp9X-siRNA (**a**) or treated with WP1130 at the concentration of 1.25 and 2.5 µM (**b**). Whole cell extracts were collected prior to Western blot analysis for ATF3, Noxa and ß-actin. Numbers shows protein quantification analyzed through ImageJ. N = 3. (**c**–**e**) Ultrastructural appearance of untreated control cells using TEM. (**f**–**h**) After treatment with WP1130 at the concentration of 2.5 µM (**f**,**g**,**h**) T265-2c cells showed extensive cytosolic vacuolization (f, red arrows) and swelling of ER (g, red arrowheads) and mitochondria (h, red arrows).

Interestingly, both Usp9X pharmacological inhibition and silencing in MPNST cells elicited a significant increase in ATF3, a typical marker of ER-stress (Fig. 5a,b) and in the pro-apoptotic BH3-only protein Noxa (Fig. 5a,b). The protein expression level of a second ER stress marker, ATF4, was similarly increased in ST88-14 cells after Usp9X disruption (data not shown). Given that ATF3 and ATF4 are downstream regulators of the ER-stress response^7^ and are capable of activating Noxa^7^, we hypothesized that cell death induced by Usp9X inhibition may in part be mediated through an ER stress response.

### Ultrastructural examination of MPNST cells following Usp9X inhibition reveals evidence of paraptosis

We used transmission electron microscopy (TEM) to determine if there was ultrastructural evidence of ER stress in MPNST cells following Usp9X inhibition. T265-2c cells were treated with vehicle or 2.5 µM WP1130 for 24 h and processed for TEM. Vehicle treated cells showed intact organelles and normal ER structure (Fig. 5c–e). In contrast, WP1130 treated T265-2c cells exhibited significant morphological changes including reduced cell size and altered cell shape (Fig. 5f). In addition, following WP1130 treatment T265-2c cells exhibited numerous small and large clear cytoplasmic vacuoles (Fig. 5f). The typical ultrastructural features of apoptosis, e.g. chromatin condensation and margination and membrane blebbing were only rarely observed in treated cells (Fig. 5f–h). Striking changes in ER and mitochondrial ultrastructure previously described in paraptotic cell death^13,14^ were seen following WP1130 treatment of T265-2c cells. Compared with vehicle treated cells, WP1130 exposed cells showed significant swelling and disruption of normal ER structure with dramatic expansion of the ER lumina (Fig. 5d and g). Similarly, in contrast with normal appearing mitochondria in vehicle treated cells, numerous mitochondria in the WP1130 treated cells were markedly enlarged, edematous, disorganized and fragmented (Fig. 5e and h). Taken together, these results suggest that WP1130 induces paraptosis^13^ in T265-2c cells.

### WP1130 inhibits MPNST tumor growth *in vivo*

**Figure 6:**
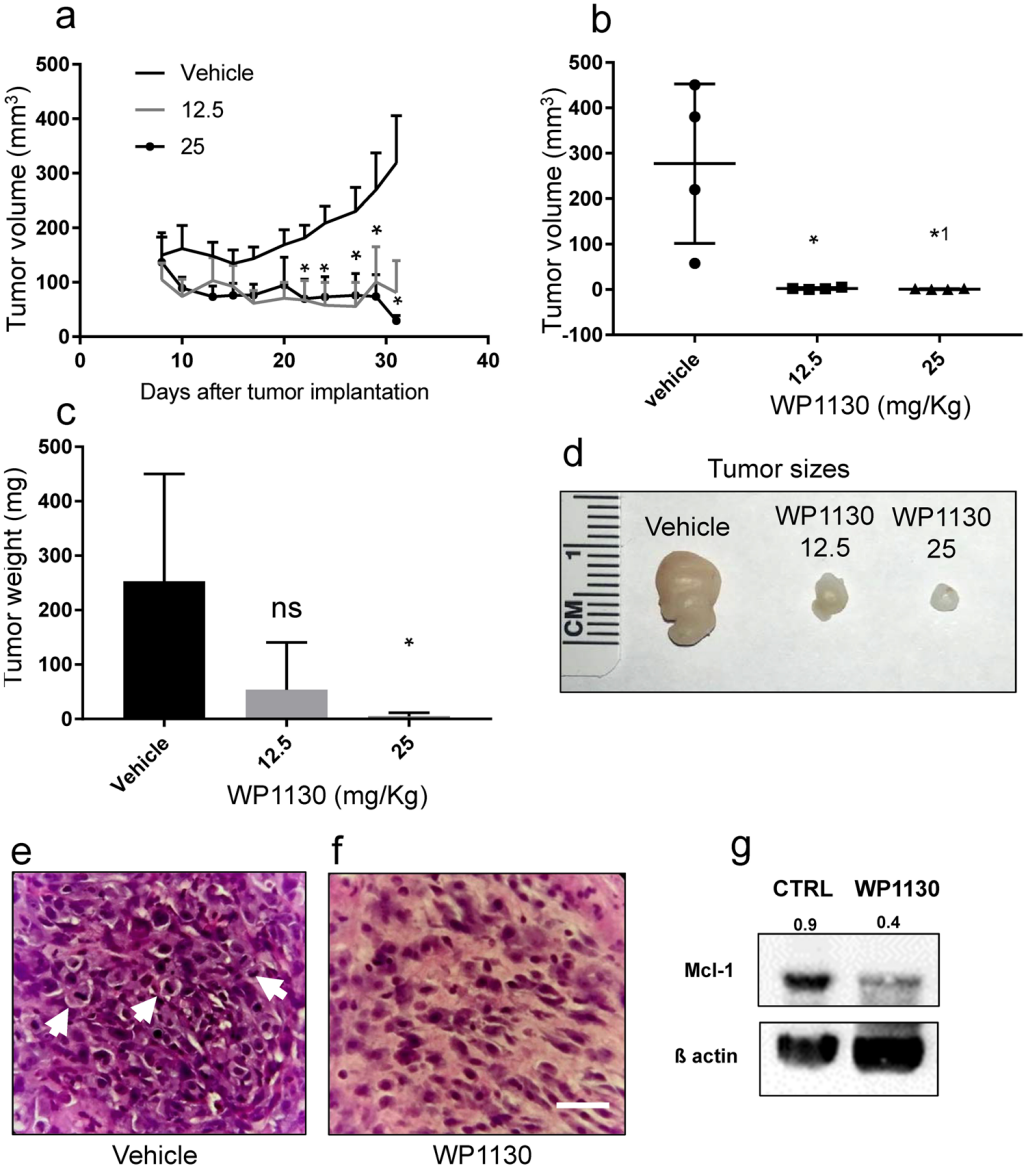
Treatment with WP1130 reduces tumor size in a mouse MPNST model generated by subcutaneous injection of ST88-14 cell line. (**a**) Tumor growth curves showing the increase in tumor size for each treatment group. Data are presented as mean and SEM. Asterisks shown only for 25 mg/Kg dose, compared to control group. Asterisks values, from left to right: 0.0413, 0.0321, 0.0409, 0.0479 and 0.0162. (**b**) Quantification of the tumors volume among different treatment groups 35 days after tumor implantation. * = 0.0202, *^1^ = 0.0198. (**c**) Quantification of the tumors weight among different treatment groups 35 days after tumor implantation. * = 0.0458. (**d**) Representative photographs of the tumors. (**e**,**f**) Representative photomicrographs showing the histological morphology (H & E staining) of tumors from mice receiving either vehicle or WP1130 at the concentration of 12.5 mg/kg. Arrows indicate mitotic figures. Scale bar, 50 µm. (**g**) Whole tissue extracts from vehicle and 25 mg/kg WP1130 treated mice were collected prior to Western blot analysis for Mcl-1 and ß-actin served as loading control. Numbers shows protein quantification analyzed through ImageJ. N = 3.

To assess the potential efficacy of Usp9X inhibition in treating MPNSTs *in vivo*, we tested WP1130 in a heterotopic xenograft MPNST model. ST88-14 cells were implanted subcutaneously and animals were divided into three groups receiving either vehicle or two different concentrations of WP1130 (12.5 and 25 mg/kg intraperitoneally) (Fig. 6 and Suppl. Figure 3). In our initial *in vivo* experiment, treatment was initiated eight days after implantation and injections were given three times/week for four weeks (Fig. 6a–f). WP1130 at 25 mg/kg per dose produced a statistically significant growth reduction with partial regression of tumors compared to vehicle treated controls (Fig. 6a). The day after the last injection, tumors were resected and the tumor volume and weight measured. WP1130 produced a significant decrease in tumor volume at both concentrations (Fig. 6b) and a statistically significant decrease in tumor weight in the 25 mg/kg dose group (Fig. 6c and d). The regression of the tumors’ size suggested that this treatment not only attenuated tumor growth but induced tumor cell death. Histopathological analysis of the resected tumors in the vehicle treated control group showed densely cellular, highly pleomorphic tumors with brisk mitotic activity (Fig. 6e). In contrast, mice treated with WP1130 showed tumors with reduced cellularity and mitotic activity, multi-focal necrotic areas and the presence of scattered apoptotic nuclei throughout the tumors at both concentrations (Fig. 6f). WP1130 inhibition of Usp9X leads to the inhibition of Usp9X function and thus, an expected decrease in the levels of Usp9X substrates^15^. To verify the *in vivo* effectiveness of WP1130 treatment, we tested the protein levels of Mcl-1 in xenografts from vehicle and WP1130 treated mice and found a significant reduction in Mcl-1 levels *in vivo* (Fig. 6g). To extend these findings, we performed a second *in vivo* experiment in which we allowed the tumors to grow to a significant size before initiating WP1130 therapy to better model the human situation. Therefore, we implanted ST88-14 cells and waited one month before initiating daily injections of WP1130 for five days per week for two weeks (Suppl. Figure 3). WP1130 treatment resulted in a significant attenuation in tumor growth when compared to the vehicle treated group (Suppl. Figure 3a). Histopathological analysis of the resected tumors showed features similar to those observed in the initial *in vivo* study. WP1130 reduced cellularity and induced cell death, with multiple areas of necrosis and presence of frequent apoptotic nuclei, at both concentrations (Suppl. Figure 3c), compared to the control group (Suppl. Figure 3b). WP1130 treatment also resulted in a marked increase in cleaved caspase 3 immunoreactivity and TUNEL labeling compared to tumors of the vehicle group (Suppl. Figure 4a–d). To preliminarily assess potential *in vivo* toxic effects of WP1130, we performed body weight measurements on vehicle and WP1130 treated animals throughout the experiment and found no differences between the three groups (data not shown) suggesting that WP1130 does not have obvious toxic effects. These results are consistent with several previous *in vivo* studies of WP1130^6,16,17^.

## Discussion

The goal of this study was to determine if Usp9X and/or Mcl-1 play(s) a role in MPNST cell survival and thus, serve as potential therapeutic targets for MPNST treatment. Usp9X is a deubiquitinating enzyme^18^ that plays a role in regulating protein degradation by modulation of ubiquitin conjugation to its targets and thereby controlling their proteasomal turnover^19^. It is well established that Usp9X plays a pivotal role in various malignant tumors affecting the nervous system, including GBM^6,11,20^. Mechanistically, Usp9X exerts its effects on cell survival at least in part by controlling the levels of anti-apoptotic Bcl-2 family members, including Mcl-1, or inhibitor of apoptosis proteins, such as XIAP^8^. Interference with Usp9X results in degradation of these survival promoting proteins^8,19^. Studies related to other malignancies, such as pancreatic cancer, demonstrated that Usp9X is necessary to drive tumor growth by inhibition of cell death^21^. However, it should be noted that the effects of Usp9X on tumors are likely to be context dependent since at least one study, involving a transgenic mouse model of pancreatic cancer, suggested that Usp9X played a tumor suppressive role^22^. Such observations do not void Usp9X as a cancer therapeutic target, but they remind us that some proteins have tumor type-specific biological roles that must be determined experimentally before advancing to human trials for any given cancer type. There might also be “context” dependent and functional differences between tumor initiation and the propagation of established malignancies. This is the first study that we are aware of examining the potential role of Usp9X in MPNST survival. Given that anti-apoptotic Bcl-2 family members are important cell death mediators in the context of MPNST and that Mcl-1 is a central regulator of apoptosis in sarcomas^10^, a pivotal role of Usp9X as a potential therapeutic target in MPNSTs appears likely.

The current study offers evidence to support the hypothesis that targeting Usp9X might significantly impact MPNST viability. In our report, by both pharmacological and genetic targeting of Usp9X *in vitro*, we detected not only inhibition of cell growth, but also massive cell death. WP1130 inhibits Usp9X deubiquitinase activity as well as that of several other deubiquitinases such as Usp5, Usp14, and UCH37^23^. First, we showed that WP1130, in a concentration- and time-dependent manner, decreases the viability of three different human NF1 patient-derived MPNST cell lines, ST88-14, T265-2c and 90-8. To determine the specific role of Usp9X in MPNST cell survival, we silenced Usp9X by siRNA. The selective knock-down of Usp9X caused a significant reduction in cell viability in all three MPNST cell lines tested. Both pharmacological inhibition and Usp9X knock down, caused time-dependent MPNST cell death with some features of apoptosis, including loss in mitochondrial membrane potential. However, the extent of caspase-3 enzymatic activity varied between cell lines being most robust in ST88-14 cells with minimal caspase activation observed in T265-2c cells. Thus, the extent of caspase-3 enzymatic activity after WP1130 in our cell lines seems to be dependent on cell-specific factors that remain to be determined. Interestingly, treatment of MPNST cell lines with a broad caspase inhibitor failed to significantly attenuate cell death after Usp9X knock-down suggesting that caspase-independent death pathways are also activated. In all MPNST cell lines tested, but most prominently in the T265-2c cell line, Mcl-1 knock-down-induced death displayed features of apoptosis such as increased annexin V staining and decreased mitochondrial membrane potential but, as in Usp9X knock-down cells, broad caspase inhibition did not protect MPNST cells from Mcl-1 knock-down-induced death. We also tested a novel small-molecule inhibitor of Mcl-1, A1210477, since these compounds are currently in preparation for human clinical application, and our results suggest that this compound exerts anti-proliferative activity against MPNST cells. From these data, we conclude that Usp9X and Mcl-1 are central cell death regulators in MPNSTs and that although caspases may be activated following their inhibition, caspase-independent pathways contribute significantly to the MPNST cell death promoting effects of Usp9X and Mcl-1 inhibition *in vitro*.

The mechanism by which Usp9X interference induced massive reduction of viability in MPNST cell lines appears to be multiple. Biochemical analysis showed that Usp9X inhibition caused an ER stress response with an increase of ATF3 and ATF4, two markers of ER stress, followed by an increase in pro-apoptotic Noxa and a subsequent decrease in Mcl-1 protein levels. Similar to our *in vitro* observations, ST88-14 xenografts following treatment with WP1130 showed a significant reduction in Mcl-1 protein levels *in vivo*.

According to previous publications^24^, upon ER stress, the ER lumen is remarkably enlarged in cells, which can be detected by electron microscopic examination. Ultrastructural examination of MPNST cells following WP1130 treatment showed features of ER stress and paraptosis, a type of caspase-independent cell death associated with ER damage. These results suggest for the first time a relation between Usp9X and ER stress in the context of MPNST. In addition, we noted an increase in pro-apoptotic Noxa, which is known to be regulated by ATF3 and ATF4^25,26^ and which preceded the down-regulation of Mcl-1^7^. Although a relationship between Noxa induction and Usp9X inhibition has not been described before in MPNST cells, these results suggest that Noxa might be implicated in the death produced by Usp9X inhibition. As explained above, we found a decline in Mcl-1 levels upon Usp9X inhibition, which is in keeping with earlier findings in other model systems^7,18,27,28^. However, our study demonstrated that Noxa up-regulation precedes Mcl-1 down-regulation, suggesting that the Noxa increase is proximal of Mcl-1 with respect to cell death induction. Given that Noxa is an inhibitor of Mcl-1 and has been known to stimulate Mcl-1 mediated proteasomal degradation, Usp9X inhibition might in part lead to a reduction of Mcl-1 levels through Noxa mediated destabilization of Mcl-1. However, taking this speculation aside, at the minimum, increased Noxa levels will facilitate release of BAK and BIM from Mcl-1, thereby driving BAX/BAK mediated intrinsic apoptosis.

In seeking to identify others forms of cell death produced by Usp9X inhibition in MPNST cells, TEM analysis showed morphological features of paraptosis with extensive cytoplasmic vacuolization, swelling of ER and mitochondria and only minimal features of apoptosis^13,14^. To the best of our knowledge, presence of paraptosis has never been described before in relation to MPNSTs or in the context of Usp9X inhibition.

To determine if Usp9X inhibition has therapeutic potential *in vivo*, we tested WP1130 in a heterotopic MPNST xenograft model. WP1130 treatment resulted in a statistically significant growth reduction and partial regression of tumors. These results suggest that WP1130 might be useful for MPNST treatment. Our findings are in keeping with the results of others that have observed anti-cancer effects of WP1130 in various xenograft model systems^6,16,17^. While we found single agent efficacy with Usp9X and Mcl-1 inhibitors in MPSNT model systems, it is tempting to speculate as to whether these effects may be further enhanced by drug combination therapies. The literature suggests that in other tumor entities, including GBM, the efficacy of Mcl-1 inhibitors is dramatically increased in the presence of other drug compounds^11,29^. Similar results have been found with regards to Usp9X inhibition^15^. For instance, Bcl-2/Bcl-xL- inhibition coupled with the inhibitor WP1130 showed synergistic anti-cancer effects in several preclinical model systems^18,30^. In conclusion, our study demonstrates that MPNST cell lines are susceptible to both Usp9X and Mcl-1 loss which appears to activate both apoptotic and caspase-independent death pathways, such as paraptosis.

## Materials and Methods

### Ethics statement

All procedures were in accordance with Animal Welfare Regulations and approved by the Institutional Animal Care and Use Committee at the Columbia University Irving Medical Center. The study was reviewed and approved by the institutional review board at the Columbia University Irving Medical Center.

### Reagents

Primary antibodies were obtained from the following sources: Usp9X (Cell Signaling, Danvers, MA #5751), Mcl-1 (Cell Signaling, Danvers, MA #5453), Noxa (Calbiochem, San Diego, CA #OP180), ATF4 (Cell Signaling, Danvers, MA #11815), ATF3 (Novus Biologicals, Littleton, CO #NBP1-85816), β-actin (Sigma, St. Louis, MO #A5316). Secondary antibodies were HRP-conjugated goat anti-rabbit (Thermo Scientific, Waltham, MA #31460) and goat anti-mouse (Thermo Scientific, Waltham, MA #31430). Degrasyn [WP1130] was obtained from Selleckchem (Houston, TX #S2243). A-1210477 was purchased from APExBIO (Houston, TX #B6011). Tunicamycin [TUN] and Staurosporine [STS] were purchased from Sigma (St. Louis, MO; TUN #T7765, STS #S5921).

### Cell culture

The human NF1-associated MPNST lines used in this study are: T265-2c cells, ST88-14 cells and 90-8 cells, obtained and maintained as described^31,32^. Morphology and doubling times of cells were routinely assessed, according to the guidelines contained in the ATCC Technical Bulletin 8. The identity of cells was verified by short tandem repeat analyses. The short tandem repeat profile of each sporadic MPNST cell line was determined by examining 15 markers standardly used by the American Type Culture Collection and the amelogenin locus (included to verify the sex of the patient from which the tumor was derived) with an AmpFLSTR system (Applied Biosystems; Foster City, CA). Cells were regularly tested for Mycoplasma infection, as well. Cells were maintained in Dulbecco’s modified Eagle’s medium (DMEM, Corning, Manassas, VA), containing 1% penicillin/streptomycin (Invitrogen, Carlsbad, CA), 1% L-glutamine (Sigma, St. Louis, MO), and 10% fetal bovine serum (FBS;Hyclone, Logan, UT) and incubated at 37 °C in a humidified 5% CO2, 95% air atmosphere. For cell viability studies, cells were plated on uncoated 96 well plates at a density of 4,000/well. For caspase 3-like enzymatic activity assays, on uncoated 48 well plates at a density of 20,000/well. For flow analyses and lysate collection, on uncoated 12 well plates at a density of 50,000/well, and in 100 mm dishes at a density of 10^6^ cells/dish. Cultures were used in experiments 24 hours post-plating unless stated otherwise. Drug treatments were performed in media supplemented with 2% FBS.

### RNAi

SignalSilence Usp9X siRNA I #6308 was purchased from Cell Signaling. Non-targeting siRNA-pool (ON-TARGETplus Non-targeting Pool, # D-001810-10-05) and Mcl-1 (SMARTpool: ON-TARGETplus Mcl-1 siRNA, L-004501-00-0005) were purchased from Thermo Fisher Scientific (Pittsburgh, PA). MPNST cell lines were transfected as previously described^33^. Cells were plated on 12 wells plates. After 24 hours, they have been incubated with the formed complexes of Lipofectamine 2000 (Invitrogen, Carlsbad, CA, USA) and the respective siRNA (12-well condition) in DMEM without FBS and antibiotics. The day after, FBS was added to a total concentration of 2%.

### Cell viability and caspase cleavage assays

Cellular proliferation was measured using CellTiter-Glo assays (Promega, Madison, WI), according to the manufacturer’s instructions. Briefly, cells were plated in 96-well plates. After treatments, 100 μl of CellTiter-Glo Reagent was added to each well containing 100 μl medium and cells. Cell lysis was induced by shaking for 2 min on an orbital shaker. Then cells were incubated for 10 min at RT for stabilization of the signal prior to measuring luminescence. Luminescence was measured using a plate reader (SpectraMax i3x multi-mode detection platform, Molecular Devices). Caspase activation was assessed with an *in vitro* caspase-3 like cleavage assay utilizing the chemical substrate DEVD-7-amino-4-methylcoumarin (AMC) (Enzo Life Sciences, Framingdale, NY #ALX-260-031). Assay was performed as previously described^34^.

### Measurement of apoptosis and mitochondrial membrane potential

For annexin V/propidium iodide (PI) staining the FITC Annexin V Apoptosis Detection Kit I (BD Pharmingen, USA) was used according to the manufacturer’s instructions. TMRE staining was performed using CellSimple™ Mitochondrial Membrane Potential Assay Kit (II) (Cell Signaling #45898), in accordance with the manufacturer instructions. Fluorescence was measured using a BD FACSCalibur flow cytometer. The data were analyzed with the FlowJo software (version 8.7.1; Tree Star, Ashland, OR, USA).

### Western blotting

Whole cell lysates were prepared by removing the media and lysing cells directly in Laemmli buffer, containing 5% 2-Mercaptoethanol (Bio-Rad #161-0710), 95% 2x Laemmli Sample Buffer (Bio-Rad #161-0737) and protease and phosphatase inhibitor cocktail (Thermo Scientific #1861281). Lysates were stored at −80 °C. Whole tumor lysates were prepared by lysing the tissues directly in RIPA buffer (Thermo Fisher #89900) with the addition of protease and phosphatase inhibitor cocktail (Thermo Scientific #1861281). Tumors were mechanically disaggregated followed by sonication. Insoluble material was removed by centrifugation at 12,000 r.p.m., and the supernatant was collected for protein quantification. Lysates were immediately stored at −80 °C.

Western blots were performed as described previously^35^. All primary antibodies were diluted to a final concentration of 1:500, except β-actin (1:8000). Secondary antibodies conjugated with horseradish-peroxidase were used at a dilution of 1:5000. For immunodetection, the Pierce ECL Western blotting substrate was utilized (Pierce ECL; Thermo Scientific, Waltham, MA). Membranes were developed using Azure c-300 gel doc & western blot Imaging (Azure Biosystem). Quantification of Western blot bands was performed with the open source image processing program ImageJ.

### Transmission Electron Microscopy

Cells were fixed with 2.5% glutaraldehyde in 0.1 M Sorenson’s buffer (PH 7.2) for at least one hour, then postfixed with 1% OsO4 also in Sorenson’s buffer for one hour. After dehydration cells were embedded in a mixture of Lx-112 (Ladd Research Industries, Inc.) and Embed-812 (EMS, Fortwashington, PA). Thin sections (60 nm) were cut on the MT-Power-Trome XL ultramicrotome. Sections were stained with uranyl acetate and lead citrate and examined under a JEOL JEM-1200 EXII electron microscope. Images were taken on an ORCA-HR digital camera (Hamamatsu) and recorded with the AMT Image Capture Engine.

### Subcutaneous xenograft model

7 × 10^6^ ST88-14 cells suspended 1:1 in Matrigel Matrix (Corning Inc., Corning, NY, USA) were implanted subcutaneously into one flank (first experiment) and into both flanks (second experiment) of 6–8 week-old SCID SHO mice as previously described^18^. 4 mice per experimental group were used. Vehicle or drug was injected intraperitoneally 3 times each week for 4 weeks during the first *in vivo* study, and for five of seven days, from Monday to Friday, for 2 weeks during the second *in vivo* experiment. For intraperitoneal application WP1130 were dissolved in a solution of 80% Cremophor EL (SIGMA, St. Louis, MO) and 20% Ethanol (Pharmco-Aaper, Brookfield, CT) (v/v), diluted 1:1 in sterile PBS.

### Histological analysis

Subcutaneous tumors and samples from organs were removed from SCID SHO mice and fixed for at least 24 h in 4% PBS-buffered formalin. Then tissues were embedded in paraffin and 4 μm thick sections were cut prior to staining with hematoxylin and eosin, TUNEL (Click-iT™ TUNEL Colorimetric IHC Detection Kit, Thermo Fisher #C10625) or cleaved caspase 3 (Cell Signaling #9661). Photomicrographs were taken at X40 and X60 magnification.

### Statistics

All data points represent mean ± S.D. All experiments were repeated at least 3 times unless stated otherwise. Representative data is shown. Statistical significance was determined by one-way ANOVA test for multiple comparisons and student’s t-test for two groups using Graphpad Prism 6 software. A p-value ≤ 0.05 was considered significant.

## Electronic supplementary material

Supplementary Figures

Original blots

## Data Availability

all the data are available upon request.
