# Supplementary material for: Usp9X Regulates Cell Death in Malignant Peripheral Nerve Sheath Tumors
Source: Sci Rep. 2018 Nov 26;8:17390. doi: 10.1038/s41598-018-35806-5 (PMC6255814; doi:10.1038/s41598-018-35806-5)
Supplement: Supplementary file 1 — Supplementary Figures [file 41598_2018_35806_MOESM1_ESM.pdf]

Usp9X Regulates Cell Death in Malignant Peripheral Nerve Sheath Tumors.

Bianchetti E, Bates SJ, Carroll SL, Siegelin MD, Roth KA

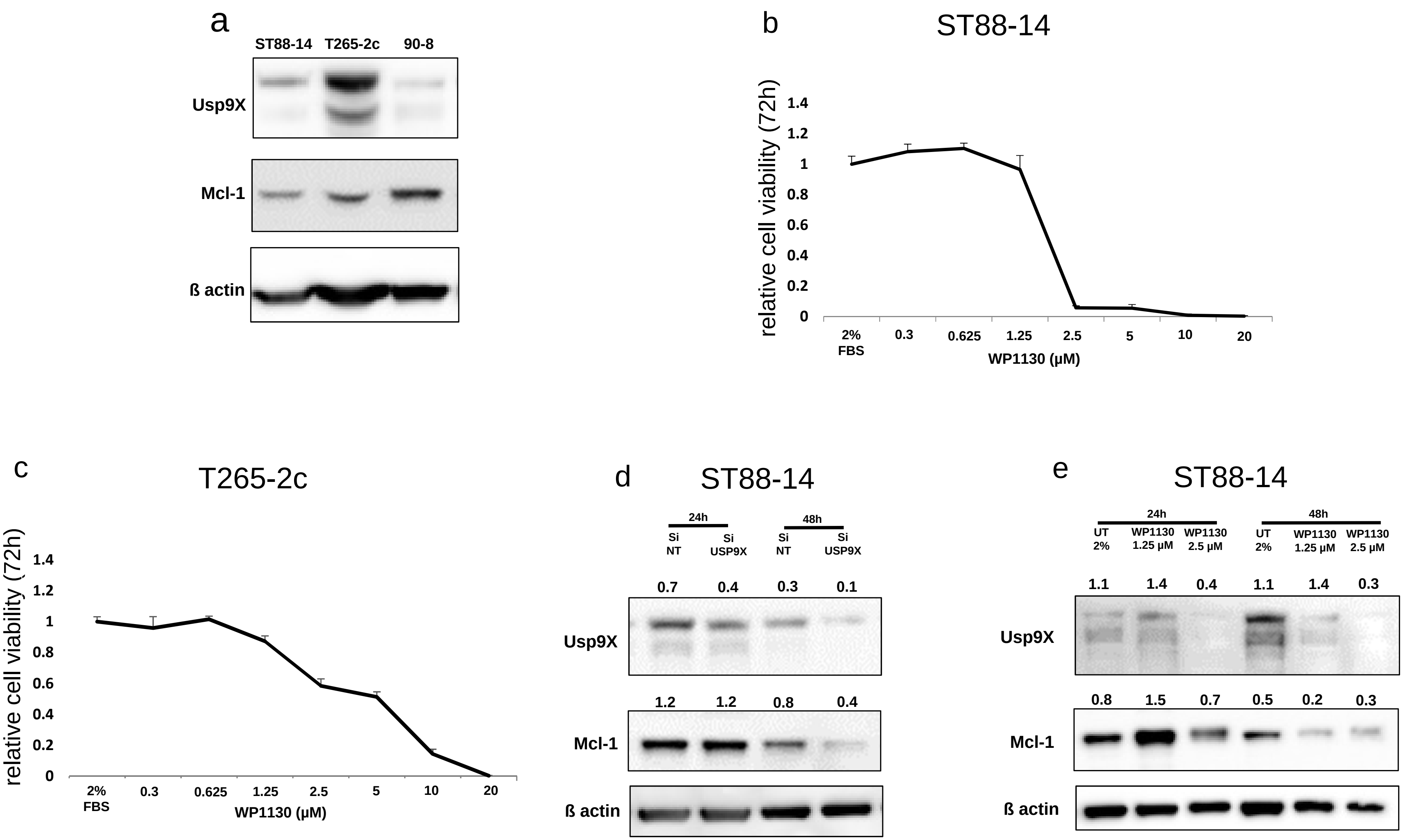
a
ST88-14
T265-2c
90-8
Usp9X
Mcl-1
ß actin
b
ST88-14
relative cell viability (72h)
1.4
1.2
1
0.8
0.6
0.4
0.2
0
2% FBS
0.3
0.625
1.25
2.5
5
10
20
WP1130 (µM)
c
T265-2c
relative cell viability (72h)
1.4
1.2
1
0.8
0.6
0.4
0.2
0
2% FBS
0.3
0.625
1.25
2.5
5
10
20
WP1130 (µM)
d
ST88-14
24h
48h
Si NT
Si USP9X
Si NT
Si USP9X
0.7
0.4
0.3
0.1
Usp9X
1.2
1.2
0.8
0.4
Mcl-1
ß actin
e
ST88-14
24h
48h
UT 2%
WP1130 1.25 µM
WP1130 2.5 µM
UT 2%
WP1130 1.25 µM
WP1130 2.5 µM
1.1
1.4
0.4
1.1
1.4
0.3
Usp9X
0.8
1.5
0.7
0.5
0.2
0.3
Mcl-1
ß actin

Supplementary Fig. 1: Usp9X expression and pharmacological inhibition in human NF1 patient-derived MPNST cell lines. Usp9X inhibition causes Mcl-1 depletion in MPNST cell lines. (a) A panel of human MPNST cell lines was analyzed for the expression of Usp9X and Mcl-1 by Western blotting. **(b, c)** ST88-14 (b), T265-2c (c) cells were treated with increasing concentrations of WP1130 for 72h. Cellular viability was determined by CellTiter-Glo® assay and the relative cell viability values were calculated normalizing data to untreated samples. Data are presented as mean and SD, n=1. **(d, e)** ST88-14 cells were transfected for 24 h and 48 h with either non-targeting (NT)-siRNA or Usp9X-siRNA (d) or treated with WP1130 at the concentration of 1.25 and 2.5 µM for 24 h and 48 h (e). Whole cell extracts were collected prior to Western blot analysis for Usp9X, Mcl-1 and ß-actin (loading control). Numbers shows protein quantification analyzed through ImageJ. N=3.

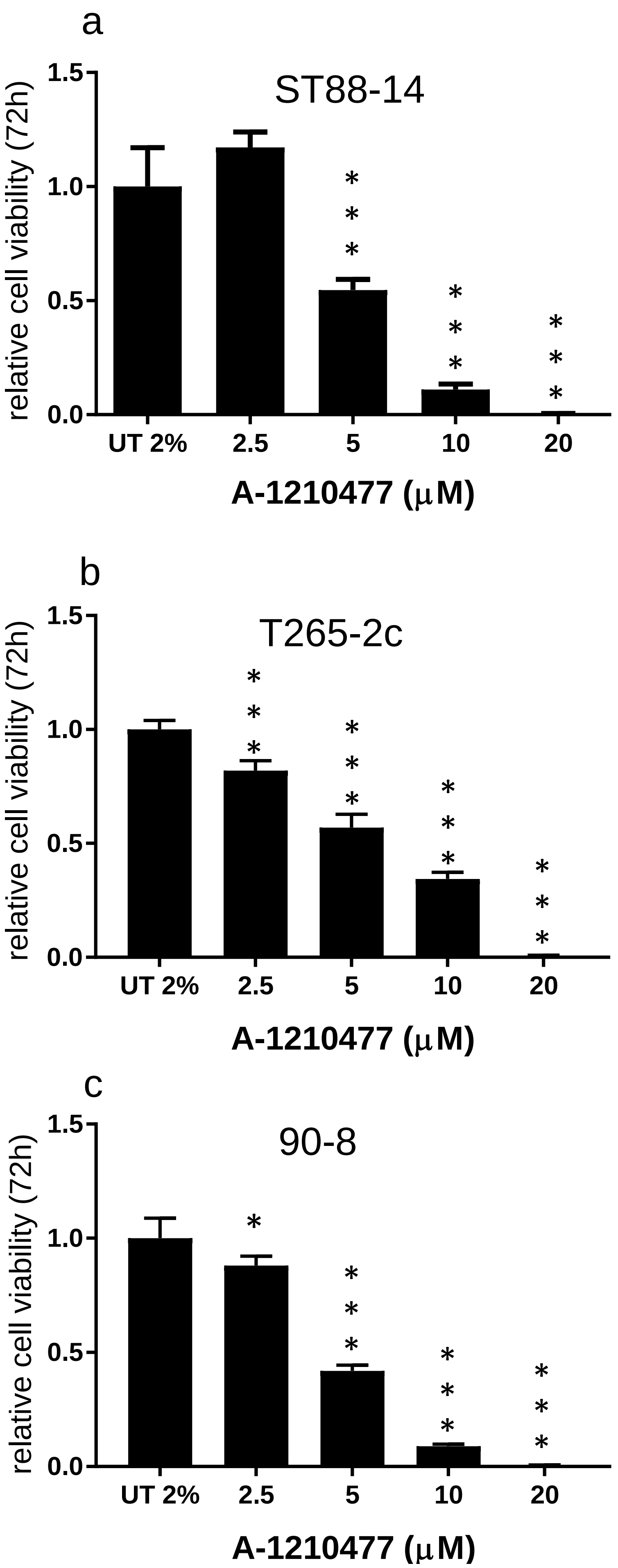

a
ST88-14
relative cell viability (72h)
1.5
1.0
0.5
0.0
UT 2%
2.5
5
10
20
A-1210477 (μM)
b
T265-2c
relative cell viability (72h)
UT 2%
2.5
5
10
20
A-1210477 (μM)
c
90-8
relative cell viability (72h)
UT 2%
2.5
5
10
20
A-1210477 (μM)

Supplementary Fig. 2: Mcl-1 pharmacological inhibition using A-1210477 reduces cell viability in MPNST cell lines. (a-c) ST88-14 cells (a), T265-2c cells (b) and 90-8 cells (c) were treated with increasing concentrations of A-1210477 for 72h. Cellular viability was determined by CellTiter-Glo® assay and the relative cell viability values were calculated normalizing data on the untreated samples. * = 0.0135, *** ≤ 0.001. Data are presented as mean and SD, n=3.

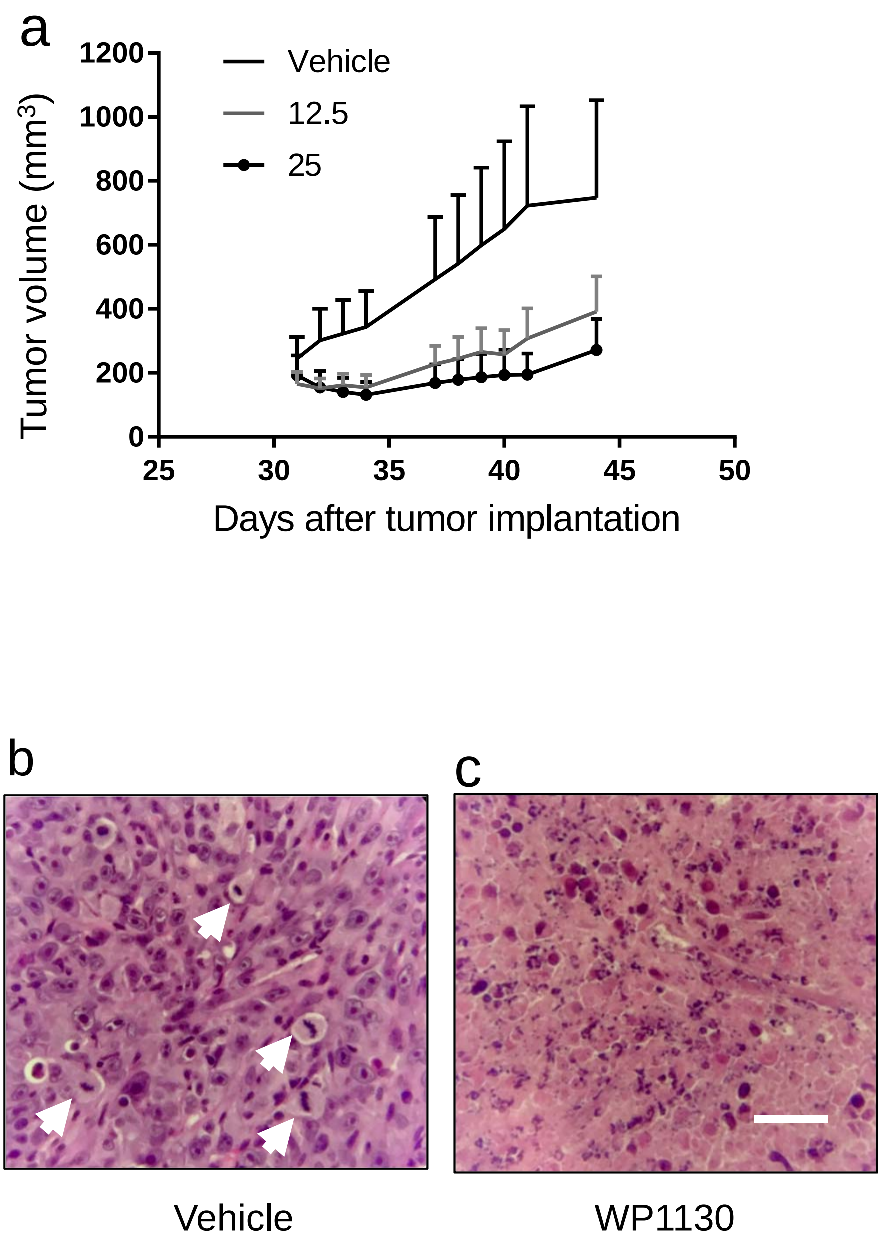
a
Tumor volume (mm3)
1200
1000
800
600
400
200
0
Vehicle
12.5
25
25
30
35
40
45
50
Days after tumor implantation
b
c
Vehicle
WP1130

Supplementary Fig. 3: Delayed treatment with WP1130 reduces tumor size in a mouse model generated by subcutaneous injection of ST88-14 cell line. (a) Tumor growth curves showing the increase in tumor size for each treatment group. Data are presented as mean and SEM. One-way ANOVA test for multiple comparisons showed a statistically significant difference between groups, with $p \leq 0.001$. **(b, c)** Representative pictures, after H & E staining, showing the histological morphology of tumors from animals receiving either vehicle or WP1130 at the concentration of 12.5 mg/Kg. Mitotic figures are highlighted by arrows. Scale bar, 50 μm.

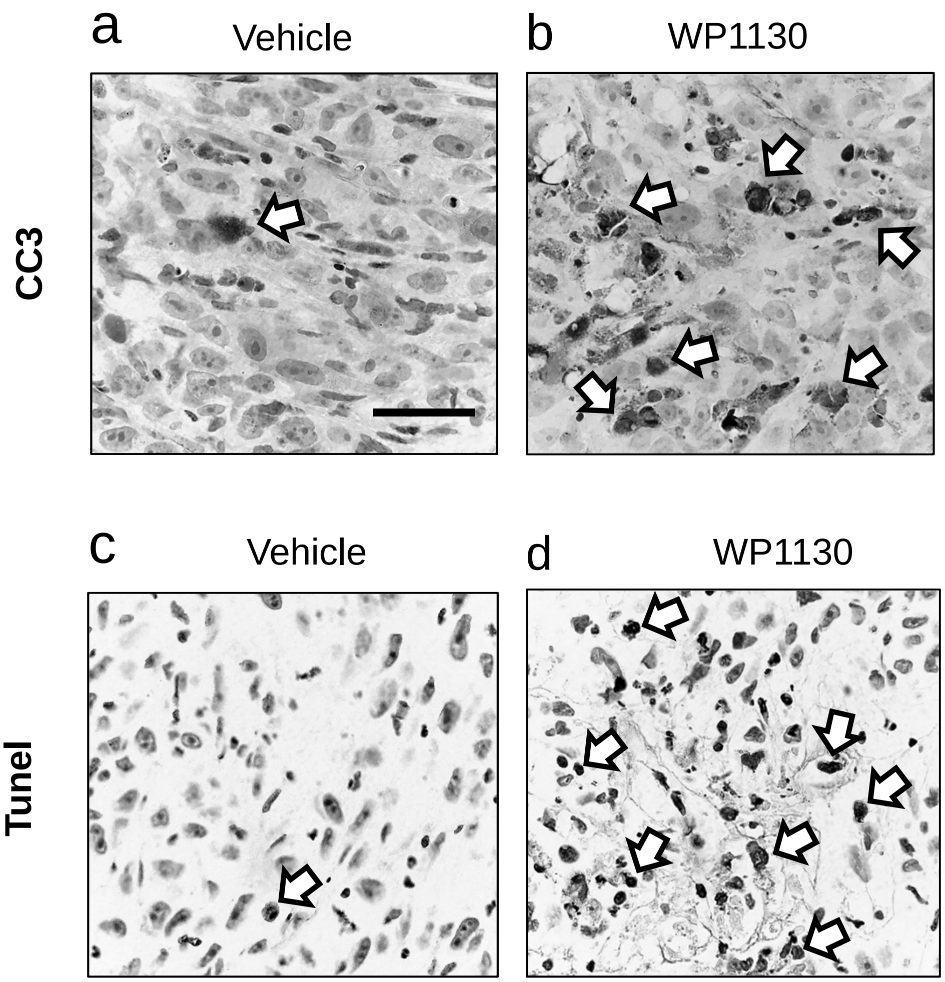
a
Vehicle
b
WP1130
CC3
c
Vehicle
d
WP1130
Tunel

Supplementary Fig. 4: WP1130 treatment *in vivo* increases cleaved caspase 3 immunoreactivity and TUNEL staining in MPNSTs. (a, b) Representative photomicrographs showing cleaved caspase 3 immunoreactivity (CC3) of tumors from mice receiving either vehicle or WP1130 at the concentration of 25 mg/kg. Arrows indicate positive cells. Scale bar, 60 µm. **(c, d)** Representative photomicrographs showing TUNEL staining of tumors from mice receiving either vehicle or WP1130 at the concentration of 12.5 mg/Kg. Arrows indicate positive cells. Scale bar, 60 µm.
