# Supplementary material for: Usp9X Regulates Cell Death in Malignant Peripheral Nerve Sheath Tumors
Source: Sci Rep. 2018 Nov 26;8:17390. doi: 10.1038/s41598-018-35806-5 (PMC6255814; doi:10.1038/s41598-018-35806-5)

Usp9X Regulates Cell Death in Malignant Peripheral Nerve Sheath Tumors.

Bianchetti E, Bates SJ, Carroll SL, Siegelin MD, Roth KA

Fig. 1 g

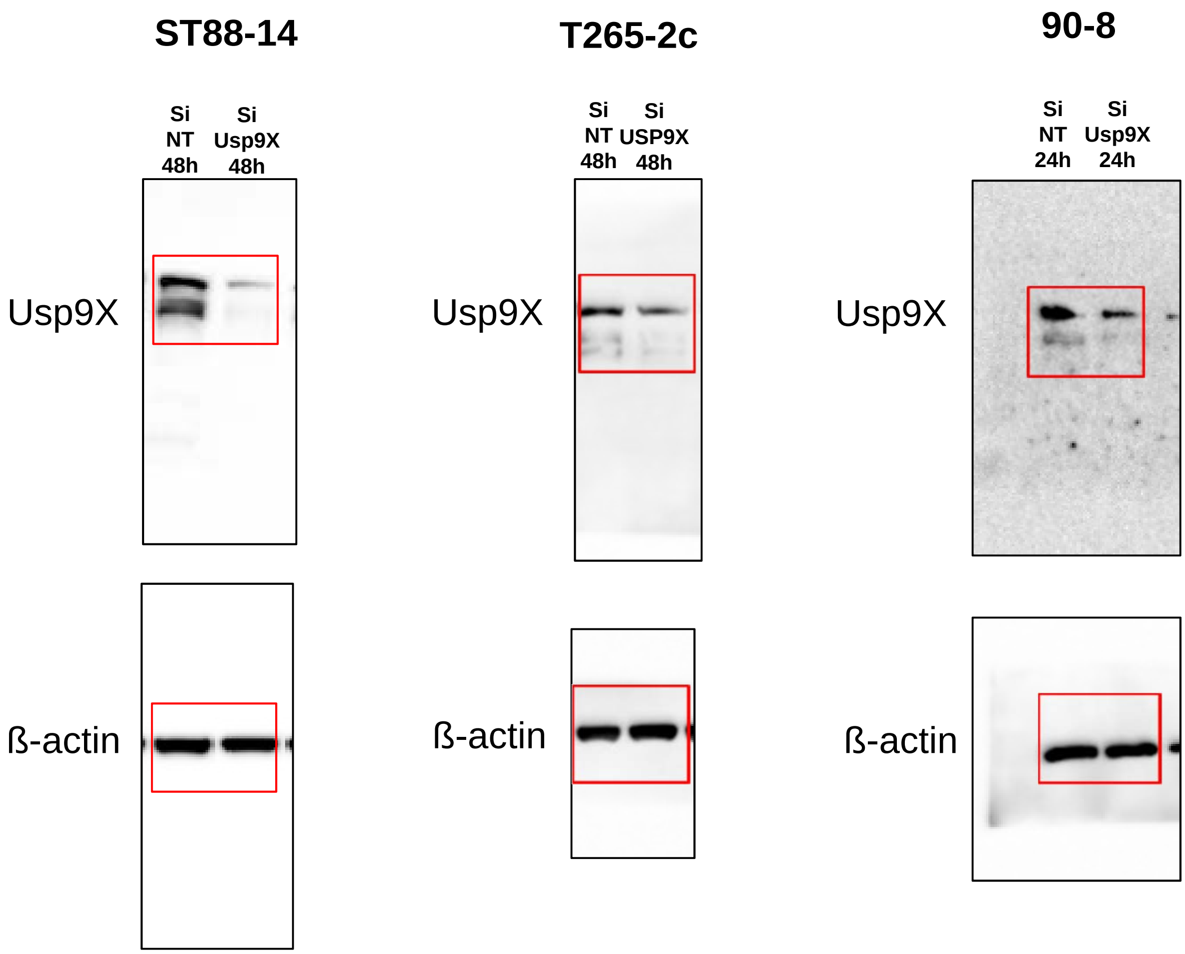

ST88-14
T265-2c
90-8
Si NT 48h
Si Usp9X 48h
Si NT 48h
Si USP9X 48h
Si NT 24h
Si Usp9X 24h
Usp9X
Usp9X
Usp9X
ß-actin
ß-actin
ß-actin

Fig. 3 h

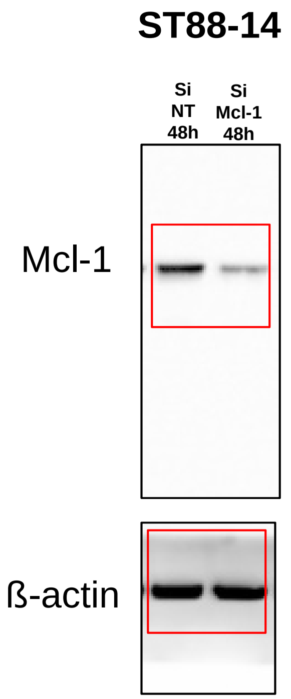
ST88-14
Si
NT
48h
Si
Mcl-1
48h
Mcl-1
ß-actin

Fig. 3 i

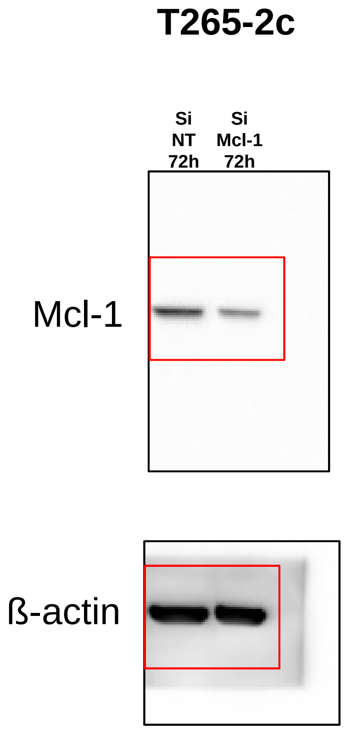
T265-2c
Si
NT
72h
Si
Mcl-1
72h
Mcl-1
ß-actin

Fig. 5 a

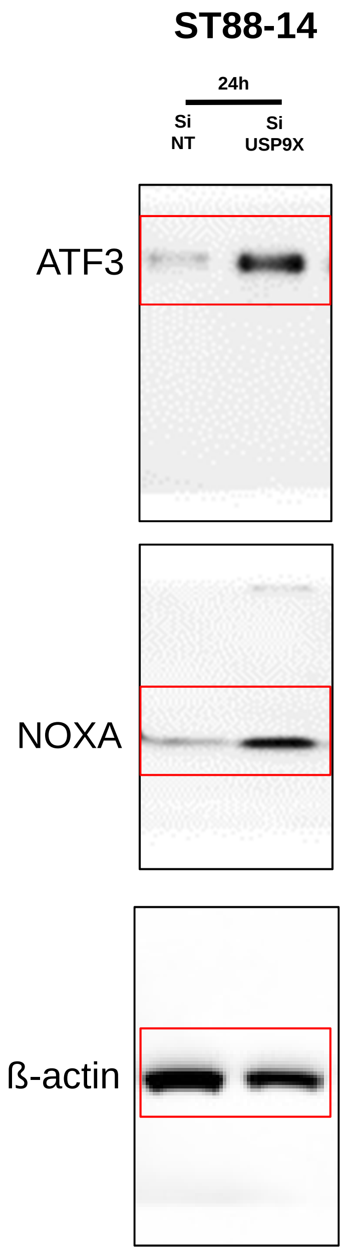
ST88-14
24h
Si
NT
Si
USP9X
ATF3
NOXA
ß-actin

Fig. 5 b

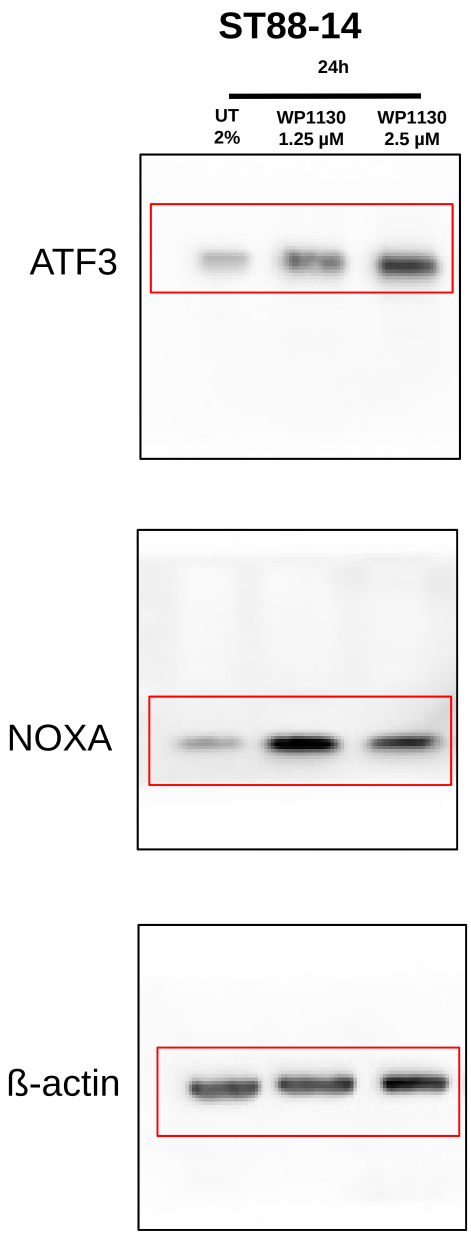
ST88-14
24h
UT
2%
WP1130
1.25 µM
WP1130
2.5 µM
ATF3
NOXA
ß-actin

Fig. 6 g

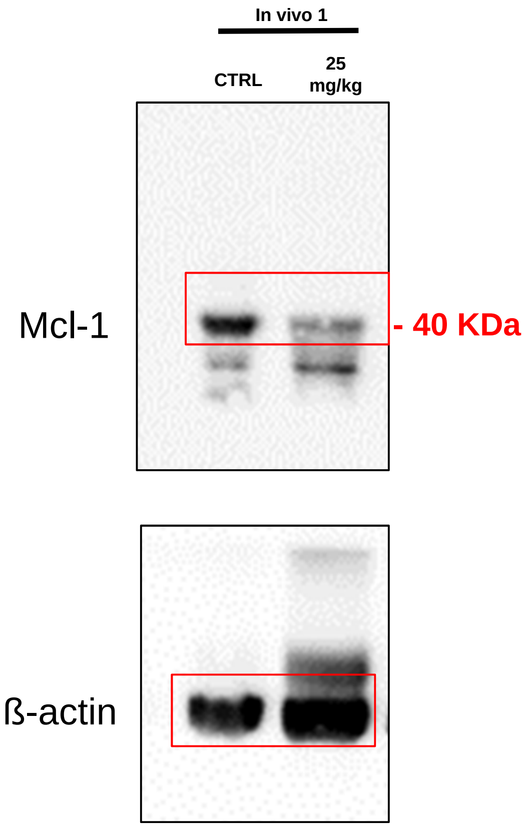

Suppl. Fig. 1 a

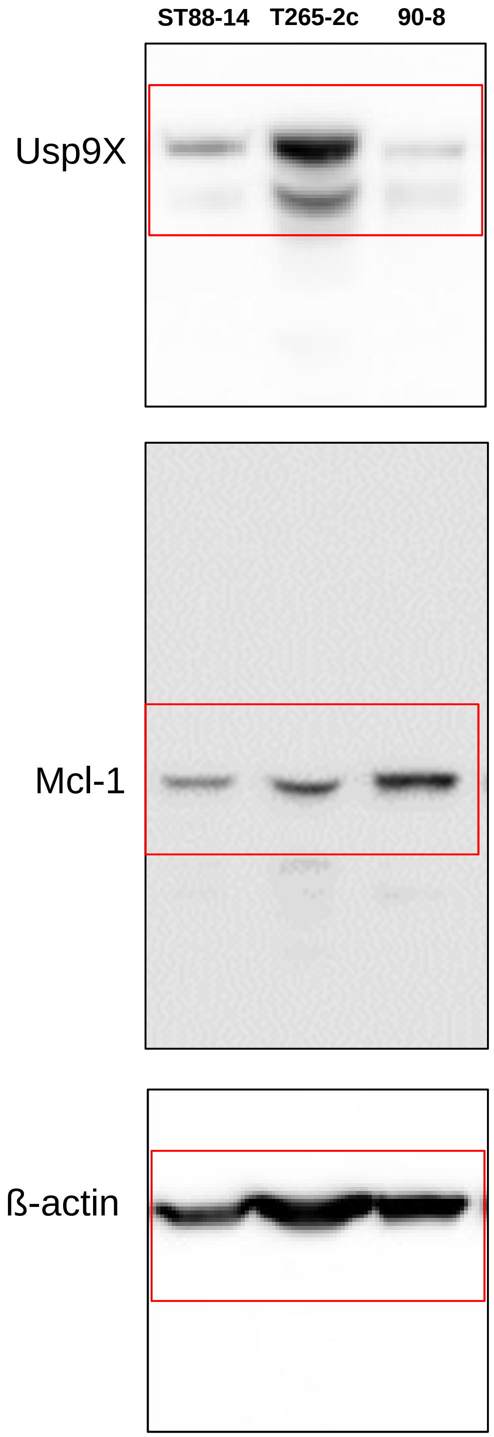

Suppl. Fig. 1 d

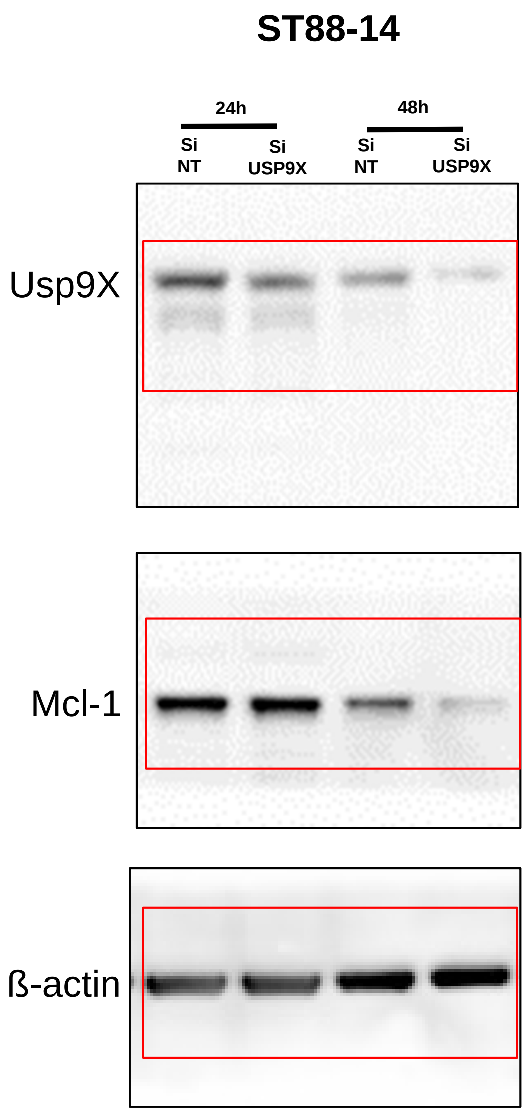

Suppl. Fig. 1 e

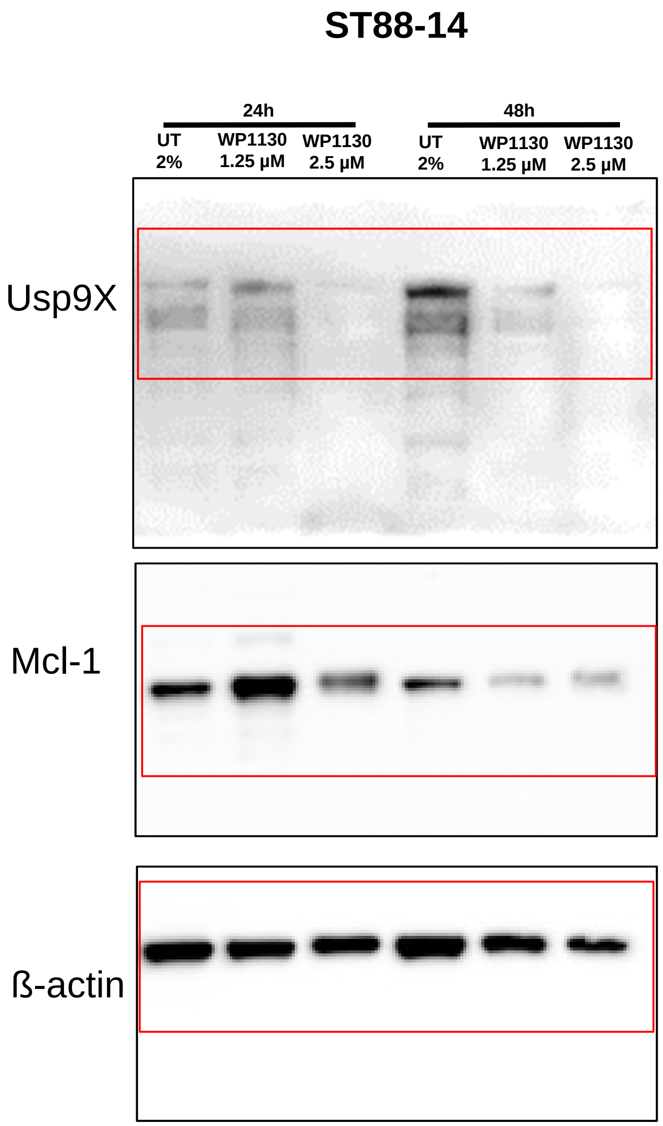

Supplement: Supplementary file 2 — Original blots [file 41598_2018_35806_MOESM2_ESM.pdf]
